# Who Needs to Save Energy and Reduce Emissions? Perspective of Energy Misallocation and Economies of Scale

**DOI:** 10.3390/ijerph20031680

**Published:** 2023-01-17

**Authors:** Weijie Jiang, Jiaying Dai, Kairui Cao, Laiqun Jin

**Affiliations:** 1 Business School, Ningbo University, Ningbo 315211, China; 2 Marine Economics Research Center, Donghai Academy, Ningbo University, Ningbo 315211, China

**Keywords:** energy-saving and emission-reducing areas, energy misallocation, economies of scale

## Abstract

With the rapid development of the economy, human survival and socio-economic development are facing the severe challenges of climate threats. Global warming is one of the greatest threats to human survival and political stability that has occurred in human history. The main factor causing global warming is the extensive use of energy; therefore, it is imperative to spend more effort in energy conservation and emission reduction. In this context, this paper provides a reference and basis for decision making on emission-reduction paths through the perspective of energy input misallocation and economies of scale of ${\mathrm{CO}}_{2}$ emissions. The results show that for cities with relatively low energy inputs, the impact of excessive energy input on ${\mathrm{CO}}_{2}$ emissions is stronger than the effect of the scale of energy input on reducing ${\mathrm{CO}}_{2}$ emissions. Therefore, these cities need to prioritize energy conservation and emission reduction. On the other hand, in cities with large energy inputs, the impact of the scale of energy input on reducing ${\mathrm{CO}}_{2}$ emissions is more significant than the effect of excessive energy input on ${\mathrm{CO}}_{2}$ emissions. Therefore, these areas should also focus on energy conservation and emission reduction. The results of this paper have theoretical value and practical significance for scientifically implementing energy conservation and emission reduction strategies, as well as reasonably planning energy conservation pathways.

## 1. Introduction

In today’s world, climate warming has become a global challenge, causing harms to the environment, health, and sustainable development, threatening people’s social development [1,2]. To address this global challenge, 175 parties signed the Paris Agreement in April 2016, and China joined it in September 2016. In September 2020, China proposed to “achieve carbon peaking by 2030 and carbon neutrality by 2060” in order to demonstrate its determination to achieve ecologically sustainable development and its commitment to building a community of human destiny. In addition, the European Union is planning that by 2030, ${\mathrm{CO}}_{2}$ emissions will be 40% lower in comparison to 1990, while the United States has proposed a 50–52% reduction from 2005 levels. Reducing ${\mathrm{CO}}_{2}$ emissions during economic development has become an urgent issue that needs to be addressed by China and the global community.

Since its reform and opening-up in 1978, China has relied on resource-intensive and polluting forms of economic development to drive rapid economic growth. [3]. Due to limitations of technical levels, the high energy consumption per unit of GDP leads to high ${\mathrm{CO}}_{2}$ emissions and increasingly serious environmental pollution [4]. By 2006, China had become the world’s largest emitter of ${\mathrm{CO}}_{2}$. Furthermore, in 2019, China’s ${\mathrm{CO}}_{2}$ emissions reached 9.83 billion tons, accounting for 29.76% of the global ${\mathrm{CO}}_{2}$ emissions. Even though the state has repeatedly emphasized paying attention to environmental protection, China is still in the process of industrialization and urbanization characterized by high energy consumption [5], so the demand for traditional energy sources is still growing. In 2020, coal consumption in China still made up a significant portion of total energy consumption at 56.8%. This highlights the pressing need for significant ${\mathrm{CO}}_{2}$ emissions reduction in the country [6]. One key step for China to achieve its carbon reduction target is to reduce energy consumption and improve the energy structure [7,8]. To reduce ${\mathrm{CO}}_{2}$ emissions, we can leverage technological innovation to enhance the efficient utilization of traditional energy sources [9] and also increase the adoption of clean energy alternatives to traditional energy sources [10,11]. Furthermore, research has shown that the development of the tertiary industry can drive down ${\mathrm{CO}}_{2}$ emissions [12], while the secondary industry increases ${\mathrm{CO}}_{2}$ emissions [13]. Therefore, we can also reduce ${\mathrm{CO}}_{2}$ emissions by changing the industrial structure and increasing the proportion of tertiary industry, such as by using internet technology to upgrade and transform industries and improve carbon emissions [14].

${\mathrm{CO}}_{2}$ emissions mainly originate from the consumption of traditional energy sources such as coal and oil. Much research has pointing out that ${\mathrm{CO}}_{2}$ emissions have a significant positive correlation with energy input [15,16]; that is, increasing energy input leads to an increase in ${\mathrm{CO}}_{2}$ emissions [17,18,19], especially in key industries, such as road transportation and construction [20]. However, there have been few studies on the changes in marginal ${\mathrm{CO}}_{2}$ emissions from energy input. Building on the existing literature, this study aims to examine the scale economic effects of energy input on ${\mathrm{CO}}_{2}$ emissions. Our empirical analysis shows that there is an “inverted-U” relationship between energy input and ${\mathrm{CO}}_{2}$ emissions. When energy input reaches a certain level, the scale economies of energy input lead to a decrease in the marginal ${\mathrm{CO}}_{2}$ emissions resulting from additional energy input. The larger the scale of energy input, the more pronounced this decrease becomes.

At the same time, China’s resource misallocation is also a very important problem. The misallocation of energy inputs significantly affects the energy efficiency of the entire industry [21,22]. Chen and Chen [23] found that among the distortion of factors affecting the efficiency of China’s resource allocation, the distortion of energy gradually exceeded the distortion of capital. This widens the gap between industries and hinders the improvement of economic efficiency [24]. At the same time, the misallocation of energy can inhibit the effective flow of production factors and upgrading of industrial structure, leading to lower efficiency in terms of carbon emissions [25]. The degree of influence is restricted by other regional factors, such as the interference of regional corruption [26]. Yang et al. [27] found that in a poorly allocated energy market, changes in pure energy and production efficiency could lead to an increase in carbon emissions. This paper further confirms that areas with excessive energy input have higher ${\mathrm{CO}}_{2}$ emissions compared with areas with insufficient energy input, and the more severe the excess, the more ${\mathrm{CO}}_{2}$ emissions there are.

This paper demonstrated, through calculation of the relevant coefficient, that areas with excessive energy input tend to have a larger amount of energy input as well. Excess energy input contributes to higher ${\mathrm{CO}}_{2}$ emissions; however, at the same time, the scale economies resulting from increased energy input can reduce marginal ${\mathrm{CO}}_{2}$ emissions. Should areas with excessive energy input (often areas with large amounts of energy input) focus on energy conservation and emission reduction, or should areas without scale economic effects (often areas with small amounts of energy input) prioritize energy conservation and emission reduction? This paper compares the perspectives of energy misallocation and scale economics and uses empirical evidence from Chinese city data to show that in areas with relatively small energy input scales, the negative effect of excess energy input on ${\mathrm{CO}}_{2}$ emissions is greater than the positive effect of scale economies on reducing ${\mathrm{CO}}_{2}$ emissions. Therefore, these areas should prioritize energy conservation and emission reduction. In contrast, in areas with relatively large energy input scales, the positive effect of scale economies on reducing ${\mathrm{CO}}_{2}$ emissions is greater than the negative effect of excess energy input on increasing ${\mathrm{CO}}_{2}$ emissions. Therefore, these areas should prioritize energy conservation and emission reduction.

In summary, the innovation of this paper lies in the fact that it differentiates the impact of energy input and the misallocation of ${\mathrm{CO}}_{2}$ emissions from the perspective of previous scholars and analyzes the impact of ${\mathrm{CO}}_{2}$ emissions from three angles: energy misallocation, energy scale economic effects, and the combined impact of energy input misallocation and scale economics. (1) From the perspective of energy scale economics, the increase in energy input brings about scale economics that reduce the marginal ${\mathrm{CO}}_{2}$ emissions from energy input, and the larger the scale of energy input, the more obvious the decrease in marginal ${\mathrm{CO}}_{2}$ emissions. (2) From the perspective of energy misallocation, areas with excessive energy input have higher ${\mathrm{CO}}_{2}$ emissions than areas with insufficient energy input, and the more severe the excess of energy input, the greater the ${\mathrm{CO}}_{2}$ emissions. (3) Considering both scale economics and energy misallocation, for areas with relatively small energy input scales, the negative impact of excess energy input on ${\mathrm{CO}}_{2}$ emissions is greater, so these areas should focus on reducing ${\mathrm{CO}}_{2}$ emissions from excess energy input. For areas with relatively large energy input scales, the positive impact of scale economic effects on reducing ${\mathrm{CO}}_{2}$ emissions is greater, so these areas should focus on reducing ${\mathrm{CO}}_{2}$ emissions from insufficient energy input.

The rest of the paper is organized as follows: Section 2 measures the degree of energy misallocation in prefecture-level cities and China as a whole; Section 3 is an empirical model design to examine the effects of energy input scale and energy allocation efficiency on ${\mathrm{CO}}_{2}$ emissions; Section 4 analyzes the empirical results and tests robustness; Section 5 selects emission-saving cities based on the above results; and Section 6 shows our conclusions and the implications of our study.

## 2. Energy Misallocation

### 2.1. Energy Misallocation Assessment Strategies

Based on the research objectives and samples, this paper aims to obtain not only the degree of loss of aggregate output value or productivity caused by the misallocation of energy inputs but also the degree of input deviation of each prefecture-level city; that is, the gap between the actual input and the effective input of each prefecture-level city. When measuring the misallocation of resources between cities, the previous research generally assumes that the overall resource input of the country is constant in the current period, and then only involves the redistribution of resources between cities. Therefore, the deviation of energy input in each prefecture-level city can be measured by the gap between the proportion of the city’s actual input to the aggregate input and the proportion of effective input. Assuming that the proportion of actual energy input in the aggregate input is $e_{i}$, and the proportion of effective input is $e_{i}^{☆}$, then the deviation degree of energy input in prefecture-level city *i* is $pe_{i}=e_{i}/e_{i}^{☆}$. The actual investment proportion $e_{i}$ can be directly obtained from the proportion of the actual input $E_{i}$ of the prefecture-level city to the aggregate investment *E*, $e_{i}=E_{i}/E$. The measurement of the proportion of input $e_{i}^{☆}$ in the effective state is the focus of this paper, and its calculation is as follows.

Referring to the practices of Brandt et al. [28] and Jin et al. [29], it is assumed that the final output of the country *Y* is the form of the CES production function of the output of $Y_{i}$ in *N* cities, $Y={\left(\sum_{i=1}^{N}Y_{i}^{\phi }\right)}^{1/\phi }$. The final outputs of the country *Y* and the city output $Y_{i}$ are both C-D production functions of capital *K*, labor *L*, energy *E*, and total factor productivity *A*, $Y=AK^{\alpha }L^{\beta }E^{\gamma }$, $Y_{i}=A_{i}K_{i}^{\alpha }L_{i}^{\beta }E_{i}^{\gamma }$. Thus, the formula for calculating the country’s productivity is:

(1) $$A={\left(\sum_{i=1}^{N}Y_{i}^{\phi }\right)}^{1/\phi }/K^{\alpha }L^{\beta }E^{\gamma }={\left[\sum_{i=1}^{N}{\left(A_{i}k_{i}^{\alpha }l_{i}^{\beta }e_{i}^{\gamma }\right)}^{\phi }\right]}^{1/\phi }$$

where $k_{i}=K_{i}/K$, $l_{i}=L_{i}/L$, $e_{i}=E_{i}/E$.

The objective function of the problem of maximizing profits from the aggregate output can be set to the following form:

(2) $$\max_{Y_{i}}\left\{PY-\left(\sum_{i=1}^{N}P_{i}Y_{i}\right)\right\}\Rightarrow \max_{Y_{i}}\left\{P{\left(\sum_{i=1}^{N}Y_{i}\right)}^{1/\phi }-\left(\sum_{i=1}^{N}P_{i}Y_{i}\right)\right\}$$

In a state of efficiency, the allocation of inputs among various elements is determined by a fully competitive market, meaning that there is no distortion or deviation in the prices of the factors of production. Thus, the goal of maximizing profits for the output of city *i* can be formulated as follows:

(3) $$\max_{K_{i},L_{i},E_{i}}\left\{P_{i}A_{i}K_{i}^{\alpha }L_{i}^{\beta }E_{i}^{\gamma }-rK_{i}-\omega L_{i}-zE_{i}\right\}$$

The first-order condition can be obtained as:

(4) $$\left\{\begin{array}{c} K_{i}={\left[P_{i}A_{i}{\left(\frac{\alpha }{r}\right)}^{1-\beta -\gamma }{\left(\frac{\beta }{\omega }\right)}^{\beta }{\left(\frac{\gamma }{z}\right)}^{\gamma }\right]}^{\frac{1}{1-\alpha -\beta -\gamma }}\\ L_{i}={\left[P_{i}A_{i}{\left(\frac{\alpha }{r}\right)}^{\alpha }{\left(\frac{\beta }{\omega }\right)}^{1-\alpha -\gamma }{\left(\frac{\gamma }{z}\right)}^{\gamma }\right]}^{\frac{1}{1-\alpha -\beta -\gamma }}\\ E_{i}={\left[P_{i}A_{i}{\left(\frac{\alpha }{r}\right)}^{\alpha }{\left(\frac{\beta }{\omega }\right)}^{\beta }{\left(\frac{\gamma }{z}\right)}^{1-\alpha -\beta }\right]}^{\frac{1}{1-\alpha -\beta -\gamma }} \end{array}\right.$$

Combined with the analysis of Dixit and Stiglitz [30], 1/(1−ϕ) also is the price elasticity of demand for various types of differentiated products, $Y_{i}=P_{i}^{1/(\phi -1)}$. Furthermore, based on $\sum_{i=1}^{N}k_{i}=\sum_{i=1}^{N}l_{i}=\sum_{i=1}^{N}e_{i}=1$, the proportion of various factor inputs in the effective state finally is:

(5) $$k_{i}^{★}=l_{i}^{★}=e_{i}^{★}=\frac{A_{i}^{\frac{1}{1-(\alpha +\beta +\gamma )\phi }}}{\sum_{i=1}^{N}A_{i}^{\frac{1}{1-(\alpha +\beta +\gamma )\phi }}}$$

Based on Equation (2), it can be seen that the proportion of factor input in city *i* in the effective state is determined by the relative size of the city’s own productivity $A_{i}$, which expresses the same meaning as the resource allocation efficiency method measured by OP covariance [31], which measures the resource allocation efficiency by the correlation between factor input share and productivity, and the higher the correlation, the higher the resource allocation effectiveness.

At the same time, the input deviation degree of various cities is $pe_{i}=e_{i}/e_{i}^{☆}$. When the index $pe_{i}$ is greater than 1, it indicates that energy input in the city is excessive, and the larger the value, the more severe the excess input. When it is less than 1, it indicates that energy input in the city is relatively insufficient, and the smaller the value, the more severe the deficiency.

Based on the C-D production function of city *i*, it can be seen that the formula for calculating the productivity of the city should be $A_{i}=Y_{i}/K_{i}^{\alpha }L_{i}^{\beta }E_{i}^{\gamma }$. In this formula, city output $Y_{i}$ should be measured in terms of aggregate output, since inputs include energy consumption as an intermediate input. However, based on the available data on Chinese cities, there are no data on total city output, only data on city GDP. Given this data deficiency, this paper corrects the production function to $A_{i}=Y_{i}/K_{i}^{\alpha }L_{i}^{\beta }$. At the same time, based on Equation (3), it can be seen that the proportion of energy input in the effective state is consistent with the proportion of labor and capital input. To this end, it can be calculated that the proportion of labor and capital input in the effective state is

(6) $$k_{i}^{★}=l_{i}^{★}=e_{i}^{★}=\frac{A_{i}^{\frac{1}{1-(\alpha +\beta +)\phi }}}{\sum_{i=1}^{N}A_{i}^{\frac{1}{1-(\alpha +\beta +)\phi }}}$$

Among them, the productivity of city *i* is obtained by $A_{i}=Y_{i}/K_{i}^{\alpha }L_{i}^{\beta }$ and $Y_{i}$ is measured by city GDP.

### 2.2. Parameter Settings and the Data

Since city fixed-asset investment data and ${\mathrm{CO}}_{2}$ data are only disclosed until 2017, this paper uses the panel data at the prefecture-level city level from 2006 to 2017. City output value $Y_{i}$ and city labor input are measured by GDP and the number of employees, respectively. The base period for the estimation of city capital stock $K_{i}$ using the perpetual inventory method is set as early as 1994; however, due to the lack of initial capital stock and fixed-asset depreciation rates at the prefecture-level-city level, this paper makes the following treatment: Firstly, this paper uses the material capital stock of each province calculated by Zhang et al. [12], and obtains the reverse deflator index of the base period according to the fixed-asset investment price index of each province. Then, we convert the material capital stock of Zhang et al. [12] with 1952 as the base period into the material capital stock with 1994 as the base period. Secondly, we calculate the capital stock of each prefecture-level city according to the share of fixed-asset investment in the base period of each prefecture-level city in the province where it is located. Thirdly, in order to obtain a more accurate depreciation rate of city fixed assets, this paper draws on the method of Ke and Xiang [32] to adjust the variable economic depreciation rate based on the fixed-asset investment structure of each city. The city energy input $E_{i}$ is measured by converting the whole city’s total amount of liquefied petroleum gas, natural gas supply, and electricity consumption into standard coal.

Referring to the α and β of factor input–output elasticity in Jin et al. [29], this paper uses the panel random-effects model of technical efficiency with time, and the α and β values are 0.32. At the same time, we use the elasticity values (α and β values are 0.27 and 0.26, respectively) estimated by the fixed-effect model and the elastic values under constant scale obtained by Brandt and Zhu [33](α and β values are 0.45 and 0.55, respectively) to test robustness. This paper refers to the practice of Brandt et al. [28] and Chen and Chen [23], assuming that the alternative elasticity of output between cities is 1/(1-ϕ) of 1.5; that is, the value of the parameter ϕ is 1/3, which is also commonly found in the literature related to real economic cycles. In the relevant trade [34], this substitution is relatively more elastic, so this paper sets the ϕ to 2/3 for robustness checking.

### 2.3. Analysis of Energy Misallocation

Table 1 shows the distribution of energy input deviation in China over the years. Furthermore, the input misallocation of various factors, as shown in Table 2. Firstly, the actual factor input ratios $k_{i},l_{i},e_{i}$ are substituted into Equation (1) to obtain the aggregate total factor productivity *A* under the actual state. Then, the proportion of various factor inputs in the effective state $k_{i}^{*},l_{i}^{*},e_{i}^{*}$ obtained in Equation (3) is substituted into Equation (1), and the aggregate productivity $A^{*}$ in the effective state can be obtained. At the same time, by assuming that only the energy input can be effectively allocated, while other factors are still in the actual distortion state, only the degree of energy misallocation can be measured. That is, in the substitution of $k_{i},l_{i,}e_{i}^{*}$ into Equation (1), $A^{E^{*}}$ is obtained; then, the degree of energy misallocation is $m^{E}=A^{E^{*}}-A$. Similarly, the degree of misallocation between capital and labor $m^{K}$ and $m^{L}$ can be calculated. As for the relevant production elasticity of the three types of factors, due to the lack of relevant data in this paper, it cannot be directly calculated. This paper mainly refers to the previous research of Chen and Hu [35] and Chen and Chen [23], and the output elasticity of capital *K*, labor *L*, and energy *E* is 0.1, 0.15, and 0.75, or 0.4, 0.32, and 0.08, respectively. In Chen and Hu’s setting [35], the output elasticity of energy input is relatively high, while the output elasticity of energy input in Chen and Chen’s setting [23] is relatively low. Table 2 shows that among the two elastic values, the energy input misallocation is the most serious of the three factors. Moreover, relative to the rapid easing of the misallocation between capital and labor, the energy misallocation has remained largely unchanged since 2011. It is enough to see that China’s energy misallocation is a very serious problem, and understanding China’s ${\mathrm{CO}}_{2}$ emission problem from the perspective of energy mismatch will also be an important issue.

## 3. Empirical Strategy and Variable Selection

### 3.1. Econometric Model Settings

Before empirically examining the influence of energy allocation efficiency on ${\mathrm{CO}}_{2}$ emissions, this paper first empirically tests the impact of energy input on ${\mathrm{CO}}_{2}$ emissions, as shown in the econometric model (7). This is used to verify the economies of scale between energy input and ${\mathrm{CO}}_{2}$ emissions; that is, as the scale of energy input increases, its marginal impact on ${\mathrm{CO}}_{2}$ emissions gradually decreases. Under the economies of scale, it is a natural conclusion that if energy saving is used to reduce emissions, the contribution of energy saving and emission reduction is greater in areas with smaller energy inputs. In the model (7), the explanatory variable *C* is the ${\mathrm{CO}}_{2}$ emissions per capita and the explanatory variable erj is the energy input per capita while controlling for city fixed effects $u_{i}$ and year fixed effects $\lambda_{t}$. The control variable *X* includes: city size csz, measured by the logarithm of the total population of the city; industrial structure ind, measured by the share of value added from secondary output in GDP; government intervention gov, measured by the share of fiscal expenditure in GDP; the level of science and technology rd, measured by the proportion of fiscal expenditure on science and technology in total fiscal expenditure; and population density pop, measured by the number of people per unit of land area in a municipal administrative area.

(7) $$\left\{\begin{array}{l} C_{it}=\beta_{0}+\beta_{1}erj_{it}+\gamma X_{it}+u_{i}+\lambda_{t}+\varepsilon_{it}\\ C_{it}=\beta_{0}+\beta_{1}erj_{it}+\beta_{1}erj_{it}^{2}+\gamma X_{it}+u_{i}+\lambda_{t}+\varepsilon_{it} \end{array}\right.$$

At the same time, this paper divides all sample cities into over-input and under-input groups by using the energy input deviation index pe. Then, it can obtain the difference in the impact of energy input on ${\mathrm{CO}}_{2}$ emission through the group regression (model (8)), so as to further test the existence of economies of scale.

(8) $$C_{it}=\beta_{0}+\beta_{1}erj_{it}+\gamma X_{it}+u_{i}+\lambda_{t}+\varepsilon_{it}\left(\mathrm{excessive}\;\mathrm{or}\;\mathrm{insufficient}\right)$$

Guided by this conclusion, this paper not only focuses on the scale of energy input but also further focuses on the efficiency of energy allocation. Then, we empirically tested the impact of energy allocation efficiency on ${\mathrm{CO}}_{2}$ emissions, reflecting on the economies of scale of energy input from the perspective of energy allocation efficiency. Additionally, we used the energy input deviation index pe to divide all sample cities into over-input and under-input groups. Furthermore, we constructed a dummy variable de, which takes the value of 1 when the energy input is excessive and 0 when the energy input is insufficient. Furthermore, we empirically tested whether areas with over-input energy have higher ${\mathrm{CO}}_{2}$ emissions than under-input areas, such as model (9). The calculation results in this paper show that only 18.5% of the cities have changed their over- or under-input state during the sample period, so the pooled OLS estimators are used to estimate it here.

(9) $$C_{it}=\beta_{0}+\beta_{1}de_{it}+\gamma X_{it}+u_{i}+\lambda_{t}+\varepsilon_{it}$$

Based on the empirical examination of the impact of energy allocation efficiency on ${\mathrm{CO}}_{2}$ emissions, and considering the differences in the impact of energy input on ${\mathrm{CO}}_{2}$ emissions between different groups, we conducted a comprehensive comparative analysis of energy allocation efficiency and energy input economies of scale. We added the energy input variable erj and its interaction with the dummy variable de×erj to model (9), forming model (10). We further constructed the interaction term pe×erj between the continuous variable of input deviation degree pe and energy input erj, forming model (11).

(10) $$C_{it}=\beta_{0}+\beta_{1}de_{it}+\beta_{2}erj_{it}+\beta_{3}de_{it}\times erj_{it}+\gamma X_{it}+u_{i}+\lambda_{t}+\varepsilon_{it}$$

(11) $$C_{it}=\beta_{0}+\beta_{1}pe_{it}+\beta_{2}erj_{it}+\beta_{3}pe_{it}\times erj_{it}+\gamma X_{it}+u_{i}+\lambda_{t}+\varepsilon_{it}$$

### 3.2. Statistical Analysis

**Figure 1:**
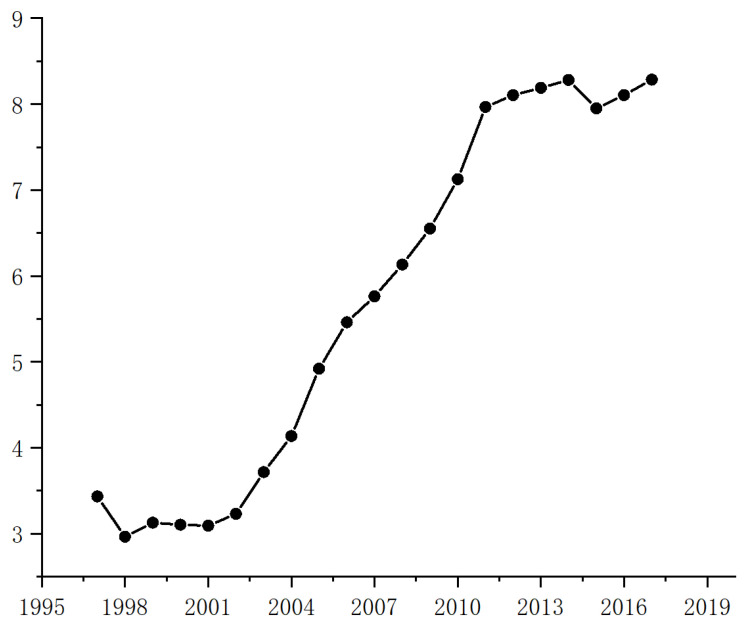
Change in ${\mathrm{CO}}_{2}$ emissions per capita.

The statistical results of each variable are shown in Table 3. Figure 1 illustrates the trend of per capita ${\mathrm{CO}}_{2}$ emissions in China from 1997 to 2017. It is evident that there has been a significant increase in emissions over this period. In 1997, per capita ${\mathrm{CO}}_{2}$ emissions were 3.47 tons, while in 2017 they had risen to 8.23 tons. This represents an average annual growth rate of 6.86%. The trend shows no sign of slowing, with emissions continuing to rise.

**Figure 2:**
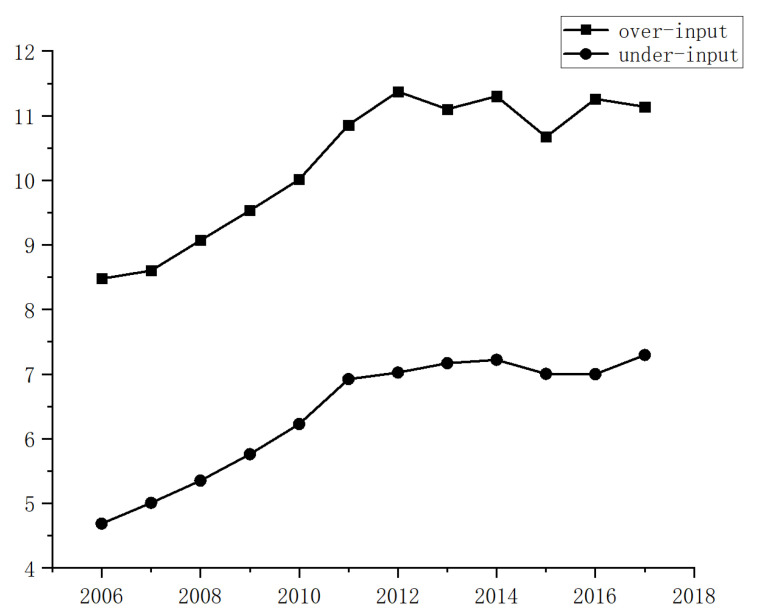
Difference in ${\mathrm{CO}}_{2}$ emissions per capita between the over-input and the under-input groups.

Then, in order to reveal the impact of ${\mathrm{CO}}_{2}$ emissions from energy input misallocation, we briefly list the difference in ${\mathrm{CO}}_{2}$ emissions per capita between the energy over-input group (de-value of 1) and the energy input under-input group (de-value of 0), as shown in Figure 2. It can be seen that the over-input cities emit on average of 4 tons more ${\mathrm{CO}}_{2}$ per capita than the under-input cities. The per capita ${\mathrm{CO}}_{2}$ emissions of China’s cities are 7.3 tons. This shows that ${\mathrm{CO}}_{2}$ emissions are much higher in cities with excessive energy input compared with cities with insufficient input. So, we should prioritize the reduction in energy inputs from over-input cities to achieve the goal of reducing ${\mathrm{CO}}_{2}$ emissions.

**Figure 3:**
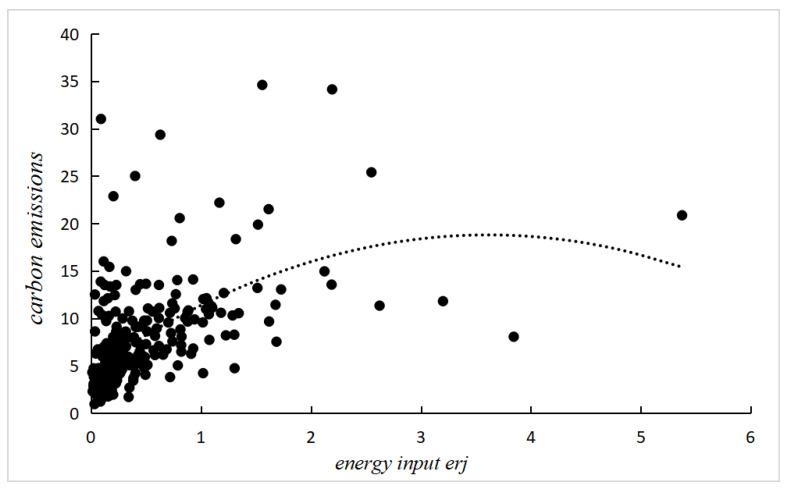
Impact of energy input on ${\mathrm{CO}}_{2}$.

A large body of literature points out that the scale of energy input can significantly affect carbon emissions. In order to clearly reveal the impact of energy input on ${\mathrm{CO}}_{2}$ emissions, we use 2017 city data to plot the relationship between energy input and ${\mathrm{CO}}_{2}$ emissions, as shown in Figure 3. We show that the two show an “inverted U-shaped” relationship, while the scale of energy input in most cities is mainly concentrated on the left side of the “inverted U-shape”; that is, with the increase in energy input, ${\mathrm{CO}}_{2}$ emissions gradually increase. At the same time, the slope of the U-shaped curve shows that the marginal ${\mathrm{CO}}_{2}$ emissions from energy inputs are decreasing. Then, we may come to a judgment: the larger the scale of energy inputs, the smaller the marginal reduction in ${\mathrm{CO}}_{2}$ emissions, and the poorer the reduction effect, while the smaller the scale of energy inputs, the greater the marginal reduction in ${\mathrm{CO}}_{2}$ emissions. The explanation for this phenomenon is that the scale of China’s energy input is closely related to the level of economic development, and the larger the scale of energy input, the higher the level of economic development of the area. On the one hand, based on the perspective of economies of scale, the process of economic agglomeration will increase the competitive pressure between firms, force firms to update technology, and then indirectly bring about a reduction in resource consumption and environmental pollution degree under the unit output of firms. On the other hand, firms may also actively carry out environmental protection and improve social reputation to enhance their differentiated competitive advantages. Moreover, economic agglomeration will also reduce the learning cost and improve the speed of information and technology dissemination between firms, thereby improving the technological progress of other firms within the agglomeration to reduce the marginal emission of ${\mathrm{CO}}_{2}$ [36,37,38,39].

In contrast, the marginal ${\mathrm{CO}}_{2}$ emissions of energy inputs are larger in areas with small energy inputs due to the limitations of a backward economic development level, backward technology level, and inadequate infrastructure.

In order to consider the impact of energy misallocation and economies of scale on ${\mathrm{CO}}_{2}$ emissions, we conducted a comparative analysis. We calculated the correlation coefficients between energy misallocation and energy input and found that there is a significant correlation; that is, the areas with excessive energy input are characterized by generally large energy input. The perspective of energy misallocation tells us that areas with excessive inputs are generally areas with large energy input and should save energy and reduce emissions, while the perspective of economies of scale based on energy input leads to the opposite conclusion: areas with low energy input are generally areas with insufficient energy input; however, they should be more energy-saving and emission-reducing. Therefore, how to balance the impact of energy misallocation and economies of scale on ${\mathrm{CO}}_{2}$ emissions is the focus of this paper.

## 4. Analysis of Empirical Results

### 4.1. The Impact of Energy Input on ${\mathrm{CO}}_{2}$ Emissions

Based on Table 4 columns (1)–(2), it can be seen that energy inputs will significantly increase ${\mathrm{CO}}_{2}$ emissions. From the fixed-effect model, a one unit increase in per capita energy input would increase ${\mathrm{CO}}_{2}$ emissions per capita by 0.298 tons. However, columns (3)–(6) show that the marginal impact of energy inputs on ${\mathrm{CO}}_{2}$ emissions tends to be decreasing ($erj^{2}$ is negative). In other words, the amount of ${\mathrm{CO}}_{2}$ increase brought about by each additional unit of energy input is decreasing. However, this result does not mean that energy input should be transferred from cities with low energy consumption to those with high energy consumption, or that cities with low energy consumption have a better emission-reduction effect than those with high energy consumption, as analyzed below.

### 4.2. The Impact of Energy Input Allocation on ${\mathrm{CO}}_{2}$ Emissions

Then, we empirically analyzed the impact of energy input on ${\mathrm{CO}}_{2}$ emissions in groups. Table 5 shows that for the excessive group, the increase in energy input does not significantly increase ${\mathrm{CO}}_{2}$, while for the insufficient group, the increase in energy input will significantly increase ${\mathrm{CO}}_{2}$ emissions. From the fixed-effect model, a one unit increase in per capita energy input would increase ${\mathrm{CO}}_{2}$ emissions per capita by 8.664 tons in the under-input group. Furthermore, this paper further empirically tested the strong correlation between the scale of energy input and the intensity of input.

### 4.3. Economies of Scale or Allocation Efficiency

Furthermore, this paper analyzed the difference in ${\mathrm{CO}}_{2}$ emissions between the over-input group and the under-input group (this is shown in Table 6). Empirical analysis based on model (9) shows that cities with excessive energy input have significantly higher ${\mathrm{CO}}_{2}$ emissions than cities with insufficient energy input. At the same time, we put the dummy variables of energy input state and their interaction with energy input into a unified empirical model and used model (10) to carry out a comprehensive analysis. The empirical results are shown in Table 7 and Table 8.

**Figure 4:**
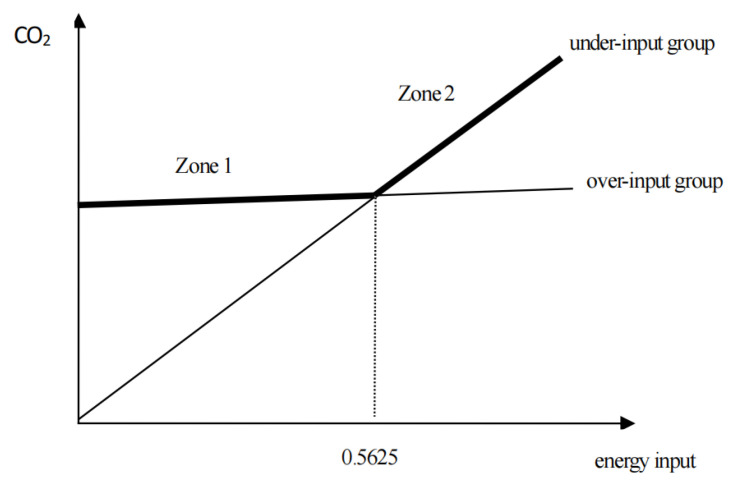
Differences in the relationship between energy input and ${\mathrm{CO}}_{2}$ in different state groups.

Based on the results in Table 7, for column (1), the regression equation for energy input on carbon emissions for the under-input group (de = 0) can be summarized as: C=9.9547erj; while the regression equation for energy input on carbon emissions for the over-input group (de = 1) can be summarized as: C=3.7639+1.9691erj. The energy input at the intersection of the two is 0.4713. Similarly, in order to improve the scientific credibility and reliability of the results, the energy input values at the intersection of the under-input and over-input groups listed in Table 5 are also obtained. The mean value of the intersection based on the various methods listed after adding the control variable is 0.5625 (as shown in column (2)–column (4)). The following will take the energy input scale of 0.5625 as the dividing line; that is, the area with an energy input less than 0.5625 is the area with a small energy input scale, and the area with an energy input greater than 0.5625 is the area with a large energy input scale. Accordingly, the regression curves of ${\mathrm{CO}}_{2}$ to energy input in the under-input and over-input group are simply obtained, as shown in Figure 4.

Furthermore, we empirically analyzed the spatial externality of the energy input using the spatial regression model, and the corresponding econometric model is:

(12) $$C_{it}=\beta_{0}+\lambda W\times C_{it}+\beta_{1}erj_{it}+\beta_{2}W\times erj_{it}+\gamma X_{it}+u_{i}+\lambda_{t}+\varepsilon_{it}$$

where *W* is the spatial 0–1 weight matrix of whether cities are next to each other. $\beta_{1}$ reflects the effect of the city’s own energy input, namely the direct effect, while $\beta_{2}$ reflects the effect of neighboring cities’ energy input, namely the indirect effects or partial spillover effect.

The direct effect reflects the impact of the energy input in city *i* on ${\mathrm{CO}}_{2}$ emissions only in city *i*, which includes the feedback effect. The spatial spillover effect reflects the effect of the energy input in neighboring city *j* on city *i*. The total effect is the sum of the direct effect and the spatial spillover effect, which can be interpreted as the average effect of the energy input in a certain city on ${\mathrm{CO}}_{2}$ emissions in all cities.

The empirical result is shown in Table 9. It can be seen that the coefficients of the spatial lags of the dependent variables are significantly non-zero (as shown in the regression results of the variable W×C), which indicates that only using the regression coefficients of the model to measure the spatial spillover effects of energy input will have systematic bias; therefore, we use the empirical results of the direct and indirect effects shown in the table to perform the analysis. As shown in Table 9, the energy input only increases local ${\mathrm{CO}}_{2}$ emissions (as shown by the direct effects). However, there is no significant spatial spillover effect (as shown by the spillover effects), which means that the energy input does not increase the ${\mathrm{CO}}_{2}$ emissions in the neighboring areas.

### 4.4. Robustness

In this paper, robustness tests are carried out by changing the alternative elastic ϕ of output between cities and the elastic α, β of factor input–output, respectively. Firstly, according to the relevant trade literature [34], we set ϕ as 2/3, and the results are shown in Table 10. Secondly, we tested the elasticity values estimated by the fixed-effect model (α value of 0.27, β value of 0.26) and the elasticity value of unchanged scale obtained by Brandt and Zhu [33] (α value of 0.45, β value of 0.55), and the results are shown in Table 11. It can seen that the results are consistent with the above, indicating that the results in this paper are robust.

## 5. The Options of Energy-Saving Cities

Based on the above results, this paper divides Chinese cities into four groups. The cities in Group (1) are those with insufficient input and energy input greater than 0.5625. The cities in Group (2) are those with insufficient input and energy input less than 0.5625. The cities in Group (3) are those with excessive input and energy input greater than 0.5625. The cities in Group (4) are those with excessive input and energy input less than 0.5625. The results are shown in Table 12.

Based on Figure 3, it can be seen that for areas with energy input less than 0.5625, the ${\mathrm{CO}}_{2}$ emissions of Group (2) are significantly better than those of Group (4). That is, in areas with less energy input, the under-input cities have less ${\mathrm{CO}}_{2}$ emissions, and the over-input cities have more ${\mathrm{CO}}_{2}$ emissions. However, for areas with energy input more than 0.5625, the ${\mathrm{CO}}_{2}$ emissions of Group (3) are significantly better than those of Group (1). That is, in areas with more energy input, the over-input cities have less ${\mathrm{CO}}_{2}$ emissions, and the under-input cities have more ${\mathrm{CO}}_{2}$ emissions.

Table 12 shows that most of the cities are concentrated in Group (2), and almost all of these cities belong to Tier 3, 4, and 5 cities (Some are Tier 2 cities), which is consistent with the regional differences in energy consumption reported by Liu [40]. Referring to the China City Statistical Yearbook and China Energy Statistical Yearbook, these cities have a more backward level of economic development, technology, and infrastructure, and their own energy output is not high. These conditions will increase a lot of costs to the energy inputs of firms, such as the cost of purchasing energy from other places and the cost of transportation, while the lack of technology and related talents will lead to low production efficiency, resulting in less output and revenue. In this case, the location of these areas does not attract a large number of energy-dependent firms, and the firms surviving in these areas may be some local firms with relatively high technical levels. At the same time, because there are not many competitors, firms can have additional funds to control ${\mathrm{CO}}_{2}$ emissions, resulting in Group (2) emitting less ${\mathrm{CO}}_{2}$ than Group (4) under the same energy input.

For Group (4), referring to the China Energy Statistical Yearbook, it can be seen that the energy output of these cities is extremely low (except for Datong, Luzhou, Mianyang, Guangyuan, Leshan, and Dazhou). Therefore, the energy used in these areas needs to be purchased from other places, resulting in a small scale of energy input in Group (4). However, most of the cities in Group (4) are concentrated around Shanxi Province. Shanxi Province is a major coal province and energy base in China, which drives the energy input of the surrounding areas. So, the surrounding areas of Shanxi Province have a larger energy input compared with the areas in Group (2). Similarly, these cities are poor in economic development, infrastructure, and technical personnel, creating low demand for energy input and allowing these areas to reach the optimal level. However, the energy input driven by Shanxi Province exceeds the optimal energy input level, resulting in the phenomenon of excessive energy input in these areas. In addition, according to the China City Statistical Yearbook, most of the cities in Group (4) have a large number of industrial firms, which exceeds the optimal number of firms. Due to the limitations in talent and technology, the competition between firms is more about price, which leads to insufficient R&D investment and no extra funds for ${\mathrm{CO}}_{2}$-emission-reduction research. Firms are more likely to choose to sacrifice the environment for maximum productivity in response to fierce competition. This competition makes Group (4) have higher ${\mathrm{CO}}_{2}$ emissions than Group (2) for the same energy input.

The cities in Group (1) are concentrated in China’s four major industrial zones (Liao-Zhong-Nan Industrial Base, Jing-Jin-Tang Industrial Base, Hu-Ning-Hang Industrial Base, and Pearl River Delta Industrial Base), which have large amounts of energy inputs. Due to the economic agglomeration effect of industrial zones, these areas are good at industrial development conditions and total factor productivity, and have high optimal energy input value. However, compared with Group (3), the cities in Group (1) are relatively less attractive to capital, firms, and technical talent due to the differences in geographic location, infrastructure, and economic levels. Referring to the China City Statistical Yearbook, the number of industrial cities and the amount of energy input in those cities is small, and most of the energy input is concentrated around 0.5625. The advantages brought by the industrial zone are not fully utilized, and the technical level is not improved, resulting in higher ${\mathrm{CO}}_{2}$ emissions compared with Group (3) at the same energy input.

The cities in Group (3) are not only concentrated in China’s four major industrial zones with superior industrial production conditions but are also mostly provincial capitals and cities with a high level of economic development, making them very attractive to capital, firms, and technical talents. This makes these areas have high total-factor productivity and optimal energy input value; however, the actual energy input is too large and still exceeds the optimal energy input value. There is a problem of excessive energy input. Referring to the China City Statistical Yearbook and China Energy Statistical Yearbook, we can find that the number of industrial firms in this group of areas is large and the energy input is significantly higher than 0.5625. At the same time, the industrial production technology and energy and environmental efficiency in such areas are higher. Therefore, the competition between firms is no longer a simple “bottom-up competition” but more likely to be a competition between technology and productivity. In this environment, the technological level of firms in the area is increasing, and the ${\mathrm{CO}}_{2}$ emissions from energy output are decreasing. The ${\mathrm{CO}}_{2}$ emissions generated by the same energy input are smaller than those in Group (1).

Based on the above analysis, it can be concluded that for areas with small energy inputs scale, the impact of excessive energy input on the increase in ${\mathrm{CO}}_{2}$ emissions is greater than the impact of the scale effect of energy input on the decrease in ${\mathrm{CO}}_{2}$ emissions, so the over-input areas will produce greater ${\mathrm{CO}}_{2}$ emissions under the same energy input. However, for areas with large energy inputs, the impact of the scale effect of energy input on the decrease in ${\mathrm{CO}}_{2}$ emissions is greater than the impact of excessive energy input on the increase in ${\mathrm{CO}}_{2}$ emissions, so the over-input areas will produce less ${\mathrm{CO}}_{2}$ emissions under the same energy input. The main reason for this difference is that the scale effect of energy input in areas with small energy input is not significant. Those areas do not have the infrastructure and technology advantages brought by economies of scale. However, it may lead to the “bottom-up competition” of firms to aggravate ${\mathrm{CO}}_{2}$ emissions. On the contrary, the scale effect of energy input in areas with excessive input scale is significant. Those areas have perfect infrastructure, excellent talents, and advanced technology brought by economies of scale, which can guide firms to reduce ${\mathrm{CO}}_{2}$ emissions while developing healthy competition. Then, for Group (2) and (4) where energy input is less than 0.5625, whether from the perspective of resource allocation efficiency or the perspective of reducing ${\mathrm{CO}}_{2}$ emissions, the energy input of Group (4) should be reduced, and the energy input of Group (2) should be increased, so as to improve the efficiency of resource allocation and reduce ${\mathrm{CO}}_{2}$ emissions, achieving a win–win situation of efficiency and green development. However, for Groups (1) and (3), which have energy inputs greater than 0.5625, from the perspective of reducing ${\mathrm{CO}}_{2}$ emissions, the energy input of Group (1) should be reduced, while the energy input of Group (3) should be increased.

## 6. Conclusions and Implications

To summarize, for cities with high energy inputs, those with low energy inputs require more energy conservation and emission reduction, as the scale effect of energy input on ${\mathrm{CO}}_{2}$ emissions is more significant than the effect of energy input misallocation. On the other hand, for cities with low energy inputs, those with high energy inputs require more energy-saving and emission-reducing measures, as the scale effect of energy input on ${\mathrm{CO}}_{2}$ emissions is less significant than the impact of energy input misallocation.

To address this issue, we can implement stricter environmental regulations, which have a strong “corrective effect” on energy misallocation and can help eliminate inefficient firms and promote the growth of more efficient ones through strict environmental compliance. However, the research found that the mitigating effect of environmental regulation on energy misallocation is more pronounced in areas with advanced emission-reduction technology compared with areas with limited emission-reduction technology. In other words, for areas with high energy investment and excessive energy input, strengthening environmental regulation can have a significant impact in promoting businesses to adopt green technology innovations and reduce ${\mathrm{CO}}_{2}$ emissions. However, in areas where energy input is small but still excessive, environmental regulation may have less of an impact on energy misallocation. In such areas, it may be more effective to eliminate industries with resource-intensive production processes, provide incentives for green innovation, increase environmental awareness among local consumers, and encourage them to engage in eco-friendly consumption behaviors to support the implementation of environmental regulations and reduce ${\mathrm{CO}}_{2}$ emissions.

Improving the ${\mathrm{CO}}_{2}$ emissions trading system can also help reduce ${\mathrm{CO}}_{2}$ emissions. Our findings indicate that the marginal ${\mathrm{CO}}_{2}$ emissions from energy inputs are relatively small in areas with high energy inputs and excessive energy inputs, so the impact of reducing ${\mathrm{CO}}_{2}$ emissions by reducing energy inputs may not be significant. However, this is more important for areas where energy input is low but still excessive. The ${\mathrm{CO}}_{2}$ emissions trading system can effectively address this issue. On the one hand, Tier 1 cities and municipalities directly under the central government have better conditions for technological innovation, industrial adjustment, and resource allocation. The ${\mathrm{CO}}_{2}$ emission trading system can significantly encourage technological innovation among businesses in these cities. However, due to the limitations of human resources and funds, the ${\mathrm{CO}}_{2}$ emission trading mechanism does not promote the technological level of relatively backward areas; however, it can also encourage them to trade ${\mathrm{CO}}_{2}$ emission indicators to developed areas, which have economies of scale, thereby reducing energy input and ${\mathrm{CO}}_{2}$ emissions. In conclusion, establishing a robust carbon trading system and increasing the transparency and availability of carbon market information can be effective in promoting the use of low-carbon technologies and management practices, ultimately leading to a reduction in ${\mathrm{CO}}_{2}$ emissions.

Additionally, the government can implement policy measures to incentivize the adoption of low-carbon technologies and the transition to low-carbon practices in key areas mentioned above by offering financial subsidies or tax incentives. Encouraging the use of renewable energy and the development of related industries is a viable option as well. At the same time, effectively attracting foreign investment and integrating business development and innovation is also an important measure. In China, areas with more open international trade generally have larger energy inputs and more severe excess input. Foreign direct investment in China is primarily concentrated in traditional manufacturing industries, which often have low technology levels and prioritize economic effects over environmental impacts when foreign investment is attracted. Therefore, China should regulate and conduct strict reviews of foreign investment, including environmental indicators in the review criteria. Additionally, China should strategically plan for foreign investment and encourage the upgrading of environmental protection technologies through foreign investment. This can aid high-pollution, high-energy-consumption enterprises in transitioning to more sustainable practices and ultimately reduce ${\mathrm{CO}}_{2}$ emissions.

## Figures and Tables

**Table 1:** Distribution of deviation degrees of energy input in China.

Year	Min	Max	25%	50%	75%	Median
2006	0.071191	3.463529	0.241692	0.465606	0.874102	0.673932
2007	0.072628	3.526458	0.24301	0.472711	0.880726	0.712359
2008	0.072267	3.441814	0.245792	0.47203	0.908433	0.707965
2009	0.076371	3.122626	0.255895	0.47164	0.880458	0.68363
2010	0.07284	3.445688	0.292237	0.518138	0.946473	0.741612
2011	0.080748	3.488423	0.317142	0.591989	1.055045	0.801603
2012	0.094328	3.594085	0.312214	0.57932	0.982766	0.808015
2013	0.076442	3.629448	0.328443	0.592682	1.036929	0.825306
2014	0.073777	3.490638	0.302262	0.567561	1.002852	0.788682
2015	0.073714	3.433368	0.297937	0.562504	1.027809	0.794995
2016	0.092228	3.633731	0.332886	0.582963	1.031188	0.818052
2017	0.090793	3.423355	0.325651	0.576027	1.010454	0.791371

Notes: Winsorized at 2.5th and 97.5th percentiles.

**Table 2:** Comparison of the degree of misallocation of various factors (%).

	Chen and Hu (2011)			Chen and Chen (2017)		
Year	$m^{K}$	$m^{L}$	$m^{E}$	$m^{K}$	$m^{L}$	$m^{E}$
2006	1.08	1.92	43.52	8.10	3.75	7.45
2007	1.06	1.89	38.68	7.79	3.71	6.96
2008	1.03	1.93	38.73	7.42	3.79	7.01
2009	0.96	1.9	40.10	6.87	3.77	7.17
2010	0.86	1.87	33.25	6.37	3.85	6.56
2011	0.88	1.8	26.78	6.09	3.69	6.01
2012	0.82	1.74	27.47	5.83	3.63	6.11
2013	0.75	1.68	27.55	5.52	3.59	6.14
2014	0.73	1.63	27.43	5.28	3.43	6.17
2015	0.67	1.5	27.12	5.07	3.27	6.17
2016	0.68	1.5	26.60	4.92	3.21	6.17

Notes: Authors’ calculation.

**Table 3:** Statistical analysis of the main variables.

Variable	Obs.	Mean	Std. Dev.	Min	Max
*Cpc*	3372	7.3260	6.7190	0.5285	76.3197
*Epc*	3336	0.4111	0.9714	0.0037	33.9659
*csz*	3372	5.8448	0.6723	2.8685	7.2689
*ind*	3341	0.4444	0.1401	0.0446	0.8440
*gov*	3360	0.1736	0.0991	0.0426	1.4852
*rd*	3371	0.0135	0.0135	0.0003	0.2068
*Pop*	3372	0.0420	0.0313	0.0005	0.2648

**Table 4:** Impact of energy inputs on ${\mathrm{CO}}_{2}$ emissions.

	(1)	(2)	(3)	(4)	(5)	(6)
	FE	RE	FE	RE	FE	RE
erj	0.2975 ***	0.3578 ***	1.1256 ***	1.3794 ***	1.0945 ***	1.2855 ***
	(0.052)	(0.052)	(0.122)	(0.12)	(0.118)	(0.118)
$erj^{2}$			−0.0282 ***	−0.0351 ***	−0.0274 ***	−0.0326 ***
			(0.004)	(0.004)	(0.004)	(0.004)
csz					−7.4195 ***	−4.7421 ***
					(0.684)	(0.389)
ind					−3.7785 ***	−2.9373 ***
					(0.609)	(0.606)
gov					−1.3985 *	−2.3317 ***
					(0.762)	(0.765)
rd					−5.6380	−7.2893 *
					(4.065)	(4.074)
pop					−33.5409 ***	−13.7563 *
					(10.340)	(7.411)
City FE	Yes	No	Yes	No	Yes	No
Year FE	Yes	No	Yes	No	Yes	No
cons	5.4007 ***	5.3832 ***	5.1728 ***	5.1021 ***	51.3525 ***	34.7213 ***
	(0.106)	(0.349)	(0.11)	(0.337)	(3.899)	(2.222)
$R^{2}$	0.2789	0.2786	0.292	0.2911	0.3403	0.3332
$F\backslash X^{2}$	98.1673	1161.4561	96.6111	1272.116	85.8683	1559.8582
*N*	3336	3336	3336	3336	3292	3292

Notes: Standard error in parentheses. * denotes *p* < 0.1, and *** *p* < 0.01.

**Table 5:** Differences in the impact of energy inputs on ${\mathrm{CO}}_{2}$ emissions between the over-input and under-input groups.

	Energy Over-Input Group				Energy Under-Input Group			
	(1)	(2)	(3)	(4)	(5)	(6)	(7)	(8)
	FE	RE	FE	RE	FE	RE	FE	RE
*erj*	0.0709	0.1255 **	0.0595	0.0717	8.1197 ***	8.2269 ***	8.6638 ***	8.7413 ***
	(0.06)	(0.06)	(0.056)	(0.055)	(0.331)	(0.316)	(0.368)	(0.347)
cons	7.964 ***	7.370 ***	59.691 ***	49.217 **	3.620 ***	3.611 ***	30.921 ***	16.264 ***
	(0.219)	(0.655)	(6.671)	(3.926)	(0.118)	(0.357)	(4.836)	(2.622)
Baseline Controls	No	No	Yes	Yes	No	No	Yes	Yes
City FE	Yes	No	Yes	No	Yes	No	Yes	No
Year FE	Yes	No	Yes	No	Yes	No	Yes	No
$R^{2}$	0.3418	0.341	0.451	0.448	0.406	0.406	0.434	0.431
$F\backslash X^{2}$	31.458	368.963	34.030	618.592	127.734	1610.912	100.300	1774.697
*N*	835	835	816	816	2489	2489	2476	2476

Notes: The Baseline Controls include *csz*, *ind*, *gov*, *rd* and *pop*. Standard error in parentheses. ** denote *p* < 0.05, and *** *p* < 0.01.

**Table 6:** Differences in ${\mathrm{CO}}_{2}$ emissions between excessive and insufficient groups.

	(1)	(2)	(3)	(4)
	POLS	POLS	FE	RE
*de*	3.9008 ***	4.0788 ***	0.1591	0.4435 **
	(0.28)	(0.26)	(0.18)	(0.17)
cons	4.6629 ***	30.3966 ***	52.9128 ***	38.4626 ***
	(0.26)	(1.75)	(3.97)	(2.29)
Baseline Controls	No	Yes	Yes	Yes
City FE	Yes	Yes	Yes	No
Year FE	Yes	Yes	Yes	No
$R^{2}$	0.08	0.34	0.32	0.31
$F\backslash X^{2}$	26.90	46.09	83.23	1401.77
*N*	3324	3292	3292	3292

Notes: The Baseline Controls include *csz*, *ind*, *gov*, *rd* and *pop*. Standard error in parentheses. ** denote *p* < 0.05, and *** *p* < 0.01.

**Table 7:** Comprehensive analysis of energy input and allocation efficiency I.

	(1)	(2)	(3)	(4)
	POLS	POLS	FE	RE
*de*	3.7639 ***	4.0166 ***	1.2851 ***	1.6227 ***
	(0.707)	(0.568)	(0.192)	(0.189)
*erj*	9.9547 ***	5.6012 ***	4.2137 ***	4.5916 ***
	(1.421)	(1.789)	(0.271)	(0.270)
de×erj	−7.9856 ***	−4.3143 **	−3.6662 ***	−3.9977 ***
	(1.575)	(1.751)	(0.249)	(0.249)
cons	3.2901 ***	20.9012 ***	42.9508 ***	30.2136 ***
	(0.297)	(2.397)	(3.865)	(2.225)
Baseline Controls	No	Yes	Yes	Yes
City FE	Yes	Yes	Yes	No
Year FE	Yes	Yes	Yes	No
$R^{2}$	0.292	0.378	0.373	0.369
$F\backslash X^{2}$	37.852	48.144	93.944	1826.036
*N*	3324	3292	3292	3292
Intersection	0.471	0.931	0.351	0.401

Notes: The Baseline Controls include *csz*, *ind*, *gov*, *rd* and *pop*. Standard error in parentheses. ** denote *p* < 0.05, and *** *p* < 0.01.

**Table 8:** Comprehensive analysis of energy input and allocation efficiency II.

	(1)	(2)	(3)	(4)
	FE	RE	FE	RE
*pe*	0.5723 ***	0.6589 ***	0.6294 ***	0.7523 ***
	(0.083)	(0.081)	(0.080)	(0.080)
*erj*	1.5031 ***	1.6737 ***	1.4958 ***	1.6487 ***
	(0.105)	(0.104)	(0.102)	(0.102)
pe×erj	−0.1985 ***	−0.2209 ***	−0.2016 ***	−0.2263 ***
	(0.016)	(0.016)	(0.015)	(0.015)
cons	4.7684 ***	4.6672 ***	51.4689 ***	34.2387 ***
	(0.128)	(0.347)	(3.823)	(2.168)
Baseline Controls	No	Yes	Yes	Yes
City FE	Yes	No	Yes	No
Year FE	Yes	No	Yes	No
$R^{2}$	0.32	0.32	0.37	0.36
$F\backslash X^{2}$	100.42	1437.85	91.14	1767.34
*N*	3324	3324	3292	3292

Notes: The Baseline Controls include *csz*, *ind*, *gov*, *rd* and *pop*. Standard error in parentheses. *** *p* < 0.01.

**Table 9:** Spatial regression model estimates for ${\mathrm{CO}}_{2}$ emissions.

	(1)
W×C	0.2983 ***
	(0.0293)
erj	0.277 ***
	(0.0181)
W×erj	0.0513
	(0.0447)
Direct effects	0.0502 ***
	(0.0162)
Spillover effects	0.0713
	(0.0539)
Total effects	0.1215 ***
	(0.0368)
Baseline Controls	Yes
City FE	Yes
Year FE	Yes
*N*	3292
$R^{2}$	0.354

Notes: The Baseline Controls include *csz*, *ind*, *gov*, *rd* and *pop*. Standard error in parentheses. *** *p* < 0.01.

**Table 10:** Robustness Test I: Substitution of elasticity estimates of output between areas.

	(1)	(2)	(3)	(4)
	POLS	POLS	FE	RE
*de*	3.4499 ***	3.2827 ***	0.5689 ***	0.7918 ***
	(0.732)	(0.547)	(0.170)	(0.170)
*erj*	12.0062 ***	7.0024 ***	1.4111 ***	1.8014 ***
	(1.226)	(1.590)	(0.269)	(0.271)
de×erj	−9.8110 ***	−5.4840 ***	−1.0830 ***	−1.4282 ***
	(1.438)	(1.534)	(0.259)	(0.262)
cons	3.1536 ***	22.8058 ***	47.3441 ***	30.6241 ***
	(0.275)	(2.364)	(4.313)	(2.542)
Baseline Controls	No	Yes	Yes	Yes
City FE	Yes	Yes	Yes	No
Year FE	Yes	Yes	Yes	No
$R^{2}$	0.2906	0.3655	0.3239	0.3179
F\X2	32.6084	38.9608	75.5583	1469.001
N	3324	3292	3292	3292
Intersection	0.3517	0.5986	0.5253	0.5544

Notes: The Baseline Controls include *csz*, *ind*, *gov*, *rd* and *pop*. Standard error in parentheses. *** *p* < 0.01.

**Table 11:** Robustness Test 2: Replacement of input–output elasticity estimates.

	Fixed-Effect Model (α = 0.27, β = 0.26)			Constant Returns to Scale (α = 0.45, β = 0.55)		
	(1)	(2)	(3)	(4)	(5)	(6)
	POLS	FE	RE	POLS	FE	RE
*de*	6.1697 ***	1.8484 ***	2.3020 ***	1.7237 ***	2.461 ***	2.173 ***
	(0.604)	(0.238)	(0.235)	(0.302)	(0.168)	(0.169)
*erj*	5.5089 ***	3.3501 ***	3.7094 ***	6.2357 ***	6.1268 ***	5.5728 ***
	(1.301)	(0.221)	(0.221)	(2.379)	(0.616)	(0.630)
de×erj	−4.572 ***	−2.905 ***	−3.226 ***	−4.488 ***	−5.917 ***	−5.313 **
	(1.297)	(0.204)	(0.204)	(2.158)	(0.607)	(0.620)
cons	19.7067 ***	43.8696 ***	30.3201 ***	22.6566 ***	53.4054 ***	38.3918 ***
	(1.987)	(3.858)	(2.211)	(2.540)	(3.867)	(2.224)
Baseline Controls	Yes	Yes	Yes	Yes	Yes	Yes
City FE	Yes	Yes	No	Yes	Yes	No
Year FE	Yes	Yes	No	Yes	Yes	No
$R^{2}$	0.403	0.371	0.366	0.343	0.352	0.345
F\X2	49.285	92.903	1820.222	38.533	85.538	1589.486
*N*	3292	3292	3292	3292	3292	3292
Intersection	1.3496	0.6363	0.7136	0.3841	0.4159	0.4090

Notes: The Baseline Controls include *csz*, *ind*, *gov*, *rd* and *pop*. Standard error in parentheses. ** *p* < 0.05, and *** *p* < 0.01.

**Table 12:** City grouping.

(1)	(2)	(3)	(4)
Karamay	Dingxi, Hechi, Zhoukou, Chongzuo	Jiayuguan	Wuhu
Jinchang	Lincang, Longnan, Huanggang, Zhaotong	Dongguan	Weifang
Panzhihua	Luliang, Siping, Shaoyang	Wuhai	Changsha
Sanya	Weinan, Pu’er, Bozhou	Shenzhen	Nanchang
Benxi	Yongzhou, Ji’an, Shanwei, Qujing,	Urumchi	Shijiazhuang
Xinyu	Huaihua, Yulin, Shangrao	Foshan	Hefei
Ezhou	Ziyang, Lijiang, Zhumadian	Baotou	Anyang
Tongchuan	Xinzhou, Pingliang, Baoshan, Yichun	Shizuishan	Fuzhou
Fangchenggang	Fuzhou, Fuyang, Nanyang	Zhuhai	Pingdingshan
Panjin	Yiyang, Suzhou, Guang’an	Zhongshan	Changchun
Liaoyang	Bazhong, Ganzhou, Xiaogan	Guangzhou	Qingyuan
Zhenjiang	Lu’an, Ulanqab, Xinxiang	Changzhou	Luoyang
Haikou	Hulunbuir, Xinyang, Hanzhong	Xiamen	Yantai
Shuangyashan	Ankang, Neijiang, Liupanshui, Yingtan	Dongying	Nanning
Tongling	Nanchong, Heyuan, Meizhou, Xianyang	Yinchuan	Harbin
Hohhot	Bose, Suizhou, Zhangjiajie, Lishui	Zhongwei	Luzhou
Dandong	Guigang, Heze, Bayannur	Yangquan	Nantong
Huzhou	Guyuan, Wuwei, Anqing, Yuncheng	Taiyuan	Handan
Zhoushan	Ningde, Maoming, Yibin, Linfen	Suzhou	Xuzhou
	Suining, Changde, Guilin, Shantou	Laiwu	Linyi
	Jingzhou, Shangqiu, Xuchang	Hangzhou	Wenzhou
	Chuzhou, Xianning, Meishan, Wuzhou	Nanjing	Baoding
	Changzhi, Qingyang, Tianshui, Xuancheng	Xining	
	Qiqihar, Shangluo, Heihe	Wuxi	
	Chifeng, Huangshan, Mudanjiang	Ningbo	
	Zigong, Jiujiang, Cangzhou, Hengyang	Daqing	
	Chizhou, Jieyang, Jiuquan, Xingtai	Yingkou	
	Liaocheng, Songyuan, Jincheng, Zunyi,	Shaoxing	
	Zhangzhou, Kaifeng, Chengde, Zhanjiang	Wuhan	
	Tai’an, Jiamusi, Nanping, Jinhua	Lanzhou	
	Dazhou, Yan’an, Puyang, Sanming	Chaozhou	
	Hengshui, Loudi, Lianyungang, Yueyang	Zibo	
	Dezhou, Anshun, Suqian, Tongliao	Anshan	
	Huainan, Jining, Quanzhou, Zaozhuang	Guiyang	
	Baishan, Yunfu, Jinzhou, Yancheng	Ma’anshan	
	Langfang, Baoji, Tonghua, Tonghua	Fushun	
	Ya’an, Shaoguan, Shiyan, Guangyuan	Huizhou	
	Longyan, Ordos, Yichun	Dalian	
	Yuxi, Bengbu, Baicheng, Zhuzhou	Jiaozuo	
	Mianyang, Jingmen, Taizhou, Zhangjiakou	Zhengzhou	
	Fuxin, Deyang, Huaibei, Yichang	Xian	
	Guests, Putian, Liaoyuan, Jixi	Binzhou	
	Hebi, Taizhou, Huludao, Chaoyang	Jinzhong	
	Yulin, Huai’an, Tieling, Hezhou	Kunming	
	Leshan, Pingxiang, Qitaihe, Wuzhong	Qinhuangdao	
	Zhaoqing, Sanmenxia, Datong, Xiangtan	Jinan	
	Jilin, Zhangye, Weihai, Jingdezhen	Rizhao	
	Baiyin, Beihai, Yangzhou, Quzhou	Chengdu	
	Huangshi, Yangjiang, Shuozhou, Hegang	Shenyang	
	Jiaxing	Tangshan	
		Qingdao	
		Jiangme	
		Liuzhou	

## Data Availability

The data presented in this study are available on request from the corresponding author.
